# ‘Doing good by proxy’: human‐animal kinship and the ‘donation’ of canine blood

**DOI:** 10.1111/1467-9566.12534

**Published:** 2017-02-06

**Authors:** Vanessa Ashall, Pru Hobson‐West

**Affiliations:** ^1^ Centre for Applied Bioethics School of Veterinary Medicine and Science University of Nottingham

**Keywords:** blood donor, pets, companion animals, veterinary, human‐animal relations, kinship

## Abstract

This article demonstrates the relevance of animals to medical sociology by arguing that pet owners’ accounts of veterinary decision‐making can highlight key sociological themes which are important to both human and animal health. Based on semi‐structured interviews, the article argues that interspecies ‘kinship’ allows for the extension of sociological claims regarding altruism, self‐interest and mutuality from human blood donation to companion animal blood ‘donation’. Furthermore, this study extends sociological understanding of the human‐animal bond by showing how the dog's status as kin meant they were expected to donate blood, and that the act of donation itself represents an important opportunity for family ‘display’. However, owners who do not or cannot donate blood themselves describe pet blood donation as an opportunity to lessen associated feelings of guilt or obligation through ‘doing good by proxy’. These findings raise critical sociological and ethical questions concerning the risks and benefits of donation, and for how we understand third‐party decision making. Finally, the article argues for the close entanglement of human and animal health, and concludes that sociologists of health and medicine should explore the radical possibility that decision‐making in healthcare more generally might be influenced by experiences at the veterinary clinic, and vice versa. (A Virtual Abstract of this paper can be viewed at: https://www.youtube.com/channel/UC_979cmCmR9rLrKuD7z0ycA)

## Introduction

Sociology is beginning to recognise the importance of companion animals (pets) for understanding health and illness (Ryan and Ziebland 2015). This builds on increasing social scientific interest in non‐human animals generally (Hobson‐West 2007), and pet animals specifically (Charles 2015). This article elucidates and extends the relevance of animals to medical sociology by arguing that pet owners’ accounts of veterinary decision‐making can highlight key sociological themes which have important relevance to both human and animal health.

After exploring current sociological understanding of human‐companion animal relationships and arguing for the entanglement of human and animal health, the article critically examines the question of motivation in human blood and tissue donation. Literature from the social sciences and medical ethics is used to show how the concept of ‘pure’ altruism has been critiqued. In short, notions of ‘impure’ altruism, self‐interest, or mutuality are potentially more helpful in recognising that human donors may anticipate, and are motivated by, actual or potential benefits to themselves alongside the notion of helping others. Our task for this article is to consider whether these kinds of themes are also relevant to an understanding of the motivations for humans presenting their dogs as blood donors and, if so, how these motivations develop our understanding of human‐companion animal relationships and the sociology of donation.

Drawing on qualitative interviews with owners of dogs who agreed to ‘donate’ their pet's blood to a canine blood bank in the UK, we argue that owners do indeed describe self‐interested motivations alongside those of altruism or ‘gift giving’. However, in our case, these themes are mediated by an intense human‐animal bond. We then move on to discuss how owners construct their animals as family members, and explore the extent to which parallels are drawn between human and animal blood donation. We consider these findings in light of previous work on ‘kinship’ and animals (Charles 2015) and the role of ‘display’ in contemporary families (Finch 2007). Finally, we show how some individuals who do not donate blood themselves present the canine blood donation experience as enabling them to fulfil their personal obligations to society. We develop the notion of ‘proxy donors’, and consider what this could mean for wider debates concerning third party donation decision‐making.

While social scientific research on companion animal blood donation is novel, this article can be situated within a broader stream of work which is starting to show the close entanglement between human and animal health. The next sections therefore explore current sociological understanding of human‐companion animal relationships and critically consider the topic of motivation in both human blood donation and associated examples of third party donation, before the world of animal blood donation is introduced. We then report and justify our methodological approach. The data analysis is structured around three primary themes: benefits for animals, shared benefits and human benefits from canine blood donation. In the conclusion we consider the implications of our findings for both the sociology of donation and our understanding of human–companion animal relationships in the health context. Finally we use this pioneering work and it's outcomes to highlight the importance of developing a new and more inclusive approach to the sociology of health and illness which is less troubled by species boundaries.

## Human ‐ companion animal relationships and the sociology of health

In this article we add to the growing body of work which explores the sociological meaning of human non‐human animal relationships. Existing work has different theoretical and empirical objectives. Post‐humanist theorists aim to destabilise the binary social categories of ‘human’ and ‘non‐human’ (Derrida 2008). Haraway (2003, 2007), for example, proposes the image of the hybrid ‘human‐animal’, where the two parties ‘become with’ one another. Latimer (2013) takes issue with some aspects of this perspective, and argues for the continued importance of difference and division between humans and animals, suggesting that we conceptualise humans as ‘being alongside’ non‐human animals.

Other authors focus more on the discursive treatment of animals by humans. The position of pets on the boundary between ‘human’ and ‘animal’, treated both as friends and commodities, has led to pet ownership being identified as a particularly useful place to examine nature/culture boundaries and ‘understand the ways in which distinctions between the animal and the human are mobilized, disrupted, crossed and re‐crossed’ (Fox 2006: 528). Fox describes how pet owners understand their pets as both ‘human’ and ‘animal’ by virtue of the similarities and differences they perceive between their pets and themselves.

That human‐pet relationships approximate, but are distinct from, understandings of a human ‘family’ where close emotional bonds between owners and their pets exist alongside an appreciation of their ‘non‐human’ qualities, is argued by Charles (2015) who frames the interspecies familial relationship as ‘kinship’. This appears to contrast with Haraway's focus on interspecies hybridity. Furthermore Charles (2015: 715) argues that ‘Rather than witnessing a new phenomenon of post‐human families, multi‐species households have been with us for a considerable length of time but have been effectively hidden from sociology by the so‐called species barrier’. In this article, we have chosen to adopt Charles's use of interspecies ‘kinship’ as a framework for better understanding the relevance of multi‐species relationships to the sociology of human and animal health. Our primary theoretical aim is not, therefore, to try and de‐centre the human, although we appreciate that alternative ways of studying this topic are possible.

On a practical level, it is also important to recognise that human and animal health care is inextricably linked; because the biological similarity between human and non‐human animals (particularly mammals) allows medical knowledge and clinical practice to be similarly applied to many different species. To give just one example, Degeling (2009) has looked at the interrelationship between fracture care in humans and animals, and reminds us that, not too long ago, the same community medical practitioner would treat both humans and non‐humans (Degeling 2009). Consequently, technology that is used in the healthcare systems of advanced industrial societies, such as chemotherapy, prostheses and mobility aids can also be used for companion animals, assuming their human owners are willing to pay. In this article we explore one fascinating and recent example of a medical technology primarily used for one species (humans) being applied to another (dogs), resulting in a new concept for animal healthcare; canine blood banking. In our data analysis we highlight how the similarities and differences between blood systems for animals and humans are central to the owners’ accounts, and how shared donation experiences appear to strengthen ‘kinship’ relationships.

The article also confirms and extends the idea that pets are crucial to their owners' health narratives. For example, Ryan and Ziebland (2015) draw on a secondary analysis of in‐depth interviews with chronically ill patients. They show how pets are described as family members who perform a vital role in the patients’ management of their disease and argue that traditionally ‘researchers may not recognise nonhuman actors as such and therefore exclude them from consideration’ (Ryan and Ziebland 2015: 69). Our own findings ultimately support this close interrelationship or entanglement between experiences of human and animal health. We therefore show the potential of research which values the connections between human and animal health, and go further by opening up the veterinary setting (see Hobson‐West and Timmons 2016) as an empirical site worthy of medical sociological analysis and of relevance to more fully understanding human healthcare.

## Motivation in human blood and tissue donation

The voluntary, unpaid donation of blood for use in the medical treatment of humans has become commonplace in the UK, with the National Blood Service (NBS) collecting donations from 1.3 million registered blood donors through mobile community collections and permanent donation sites across the country (NBS 2016, 2016). The blood donations are processed into blood products for use in medical treatment and surgery. Interestingly, these modern day clinical practices developed from initial experiments conducted at the Royal Society in 1667, where blood was transfused directly from one dog to another (AABB 2016), meaning that the aforementioned entanglement of human and animal health science in this context is not a recent phenomenon.

The National Blood service claims that 25 per cent of us will need a blood transfusion at some point in our lives and yet just 3 per cent of the adult population in the UK are donors (NBS 2016, 2016). The constant need to increase the donor pool has made understanding the reasons why people might choose to donate or not to donate blood an important area for study. Titmuss's book *The Gift Relationship* (1970) is regarded as a seminal work which explores the social organisation of health services by citing policy statistics and empirical data relating to the donation of blood in different countries. Titmuss (1970) has been credited with highlighting the significance of altruism as a motivation for blood donation and consequently framing the donation experience as a ‘gift’. His work aimed to influence the organisation of blood collection programmes and he promoted institutional altruism; that is to say systems which depend on voluntary giving, his central argument being that altruism is both morally sound and economically efficient. Titmuss’ work has become crystallised over time and has heavily influenced both academic and policy considerations of motivation in blood donors. In more recent years, however, Titmussian ‘gift’ arguments have been increasingly criticised by social scientists who use empirical studies to identify a more complex set of motivations in donors, such as self‐interest or even guilt. Consequently, even where donors are not financially rewarded, a concept of ‘mutuality’, which recognises that donors as well as recipients stand to benefit, is being increasingly recognised.

Oakley and Ashton (1997: 8) sum up some of this criticism as indicating that Titmuss was ‘misunderstanding the anthropological literature in casting the giving as somehow essentially virtuous’. More recently, Busby (2004) explored the practical changes which have occurred since the creation of the first national blood bank in a time of post‐war solidarity. Busby uses interview data from National Blood Service donors to reinforce her argument that mutuality, not one‐directional altruism, was implied by the blood donors themselves. Furthermore, Busby claims that Titmuss's original work identified more with the theme of mutuality than is generally credited (Busby 2004).

Other work confirms that blood donor motivations go beyond altruism. After reviewing the literature, Farrugia *et al*. (2010) point to upstream and downstream reciprocity, where the donation represented gratitude for blood received by loved ones, or was intended to ensure an adequate supply for the donors’ future use. Other forms of intrinsic motivation were also reported such as an enhanced feeling of self‐esteem. Similarly, findings of a systematic review (Bednall. *et al* 2013) point to a multitude of factors, including feelings of satisfaction and a desire to avoid regret. Furthermore, this review suggested that while the first blood donation offers relief from an obligation to donate, this motivator is less reported by frequent donors. As we discuss below, our own work shows that when this perceived obligation to donate (human) blood is not satisfied, associated feelings of guilt or regret can reportedly influence the decision to offer a pet as a blood donor.

Recent research on related topics confirms the need to go beyond assumptions of altruism, including work on organ donation (Burnell *et al*. 2015, Moorlock *et al*. 2014, Shaw 2015), bone marrow donation (Garcia *et al*. 2013) and the donation of tissues to biobanks (Locock and Boylan 2016), to the point where some have concluded that the term ‘gift’ offers little utility in representing donors’ views and its use should be discouraged (Locock and Boylan 2016).

One of our tasks in this article is to consider the extent to which these findings have resonance in the case of animal blood donation. However, we also need to recognise an additional layer of complexity; namely, that in the case of animal blood donation, the individual making the donation decision is not same individual who will actually undergo the procedure. How should we therefore understand the nature of such proxy or third party donation decisions?

Human blood donations are not taken without individual consent and living donations of other tissues which are consented to by a third party are currently uncommon in human medicine (although some have recently argued that it may be justifiable to use children or ‘incompetents’ as organ donors as it may be in their best interests to donate (Wilkinson 2011; Van‐Assche *et al* 2014)). However, one potentially comparable existing area is bone marrow donation by children, generally for the treatment of family members. In medical ethics it has been suggested that taking donations from those who cannot legally consent is only justifiable if the donor themselves benefit (Spital 2004). So, does helping a family member always constitute such a benefit? Even leaving aside the physical risks of donation, this is far from straightforward. For example, donation may carry social risks for the child, if the family focus were to shift significantly from the donor to the recipient, or the donor might feel personally responsible if the donation did not go well (Bendorf and Kerridge 2011). While donation in this context could strengthen family ties (an issue we return to later), child donors may experience pressure from family or wider society to donate (Garcia *et al*. 2013).

Furthermore, the language of non‐consenting donors ‘benefiting’ or reference to prioritising the interests of ‘the donor’ (Wilkinson 2015) is considered problematic by those who argue that this is to take an individualistic approach to donation ethics. For example, in discussing the topic of so‐called ‘saviour siblings’, where children are created with compatible tissue for donation to sick family members, Taylor‐Sands (2015) argues that a more relational approach is needed, where the interests of a family unit as a whole are considered. This shift towards the family as a unit of analysis has important implications for empirical work on donation and has some similarities with our focus on interspecies families and the model of ‘kinship’ we have identified. We return to this point in the conclusion. The article now moves on to focus on the core topic of companion animal blood donation.

## Canine blood banking and the animal ‘donor’

The provision of blood and blood products to animals has become a part of contemporary veterinary practice, particularly for companion animals such as dogs and cats (Day and Kohn 2012). Blood for direct transfusion or for the preparation of veterinary blood products must be collected from another animal of the same species. One to one direct transfusions between animals were the only possible treatment option in the UK until a legislative amendment in 2005 provided a route for the larger scale production and banking of animal blood products for general therapeutic use (RCVS 2014). In contrast to human medicine, therefore, veterinary transfusion medicine is still a very young field; however, the larger of the two canine blood banks now operating in the UK has already enlisted over 7,000 canine ‘donors’ for blood product production. These animals are pet dogs or retired greyhounds awaiting rehoming whose owners offer them as ‘donors’ (PBB 2014).

The process of canines donating blood is practically quite similar to that for humans; a human collection bag is used to collect 450ml of blood from dogs weighing over 25kg. The dogs are not usually sedated but must remain in a lying position for approximately 5 minutes while the blood is collected by trained staff under the supervision of a veterinary surgeon. The risks to donors from donating blood in this manner appear to be very slight with bruising, bleeding and faintness occurring rarely, as for human donors (Day and Kohn 2012). Once collected the blood is typed and processed into plasma, red blood cells and other blood products and stored at the blood bank. Unlike human blood products in the UK, canine blood products are not publicly owned but must be paid for by the owners of the transfusion recipient. Veterinary professionals administering the transfusion must also charge for their time, and so access to animal blood products may be restricted on a financial basis. The regulation of animal blood banks encompasses governance of both professional standards (by the Royal College of Veterinary Surgeons) and product quality, with animal blood products being produced according to a licence authorised by the Veterinary Medicines Directorate.

Within the specific context of living animals classed as companions and the quasi‐medical process of collecting and banking blood, the term ‘blood donor’ is commonly used (Day and Kohn 2012). However, the use of this term for animals raises some interesting social and ethical questions, because of the assumed association with concepts of choice and consent. Recent arguments have been made against using the term ‘donor’ to describe non‐consenting humans who it is argued should be termed the ‘tissue source’ (Van‐Assche *et al*. 2014). Indeed the advent of animal ‘donors’ has already contributed to discussion over whether it is the consenting party or the tissue source who is actually ‘donating’ in both human and animal contexts (Machin and Cherkassky 2016). In short, the association between voluntary decision‐making and the concept of ‘donation’ is threatened in the veterinary context, where all ‘donors’ lack the capacity to consent.

With regards to decision‐making in companion animal medicine, a triadic relationship exists between the animal, owner and vet. This makes veterinary medicine ethically complex, especially when the welfare needs of the animal and the wishes of the owner come into conflict. In fact, the tension that veterinarians experience in trying to serve the interests of both animal patients and paying clients has been called the fundamental question in veterinary ethics (Morgan and McDonald 2007). The sociological exploration of these tensions is as yet an embryonic field but work is starting to emerge which illuminates this complex area (Morris 2012). In any case, we would highlight that this issue is also not straightforward for some human medical fields, for example in paediatrics (Dimond 2014). In the paediatric case, a triadic relationship also exists between parent, child and healthcare professional, and some authors have called for more research on this dynamic (Gabe *et al*. 2004). If we accept the notion of interspecies ‘families’ then data such as that analysed here may further illuminate professional and familial tensions which connect both veterinary and human donation practices.

In this article we will highlight how the practical similarities between human and animal blood donations, and the position of companion animals within some human families, allows us to apply the identified sociological themes of altruism, self‐interest and familial relationships beyond human medicine and into the veterinary world. Our analysis of data generated in this unique setting contributes to sociological understandings of human‐animal relationships and serves as a powerful example of the ethical and professional tensions created by the topic of third party donation decisions.

## Research methods

The lead author carried out interviews with 21 dog owners during 2014 at two canine blood collection clinics, one in the south of England and one in the Midlands. Access to the donation sites was agreed by a key contact at the canine blood bank. The project was funded through a Wellcome Trust fellowship and the project received ethical review from the University of Nottingham. Interviewees provided written consent and were assured that their names would not be used.

All interviewees were female. This interesting gender bias may partly echo the fact that women tend to attend veterinary appointments. The interviewees had varying degrees of experience in donating their pets’ blood, with some presenting for the first time and others having made numerous ‘donations’.

The interviews were semi‐structured, with open questions being used to encourage the interviewees to talk about their experiences in their own words. The interviews were conducted in the post‐donation area after the dogs had left the donation room. This strategy had the advantage that interviews occurred close in time and space to the actual donation process, but without interrupting it. This worked well and gave access to a good number of interview candidates. The disadvantage was that the time available for interview could not generally exceed 20 minutes, as determined by the need of the owners to look after their animals and leave the waiting area. The original plan (partly inspired by Busby 2010) had been to follow up these interviews with longer discussions for a sample of owners at a later date. However, this plan was revised once it became clear that the data generated through these first sessions was remarkably rich and informative, with recurrent and consistent themes being clearly identifiable.

Interviews were recorded and transcribed in full before manual coding and thematic analysis (Bryman 2012). While the identification of themes within qualitative data has long been recognised as part of other analytic methods such as grounded theory, thematic analysis is now starting to be recognised as a stand‐alone technique (Braun and Clarke 2006). Themes were drawn from the coded data by a combination of inductive and deductive techniques meaning that previously identified areas of interest such as ‘altruism’ were explored alongside emergent themes such as ‘pets as family members’. While well aware of the literature on interviews as accounts (Dingwall 1997), the approach for this particular article is to take a more literal interpretation of the data, as access to individuals’ own motivations and explanations for supporting the canine blood bank.

As with any study, the chosen methodological approach has limitations and tensions. First, owners who do not donate are left out of this process. However, this is justified by our primary desire to focus on motivation for donation, and also that we are working in an empirically novel area: it is important to start somewhere. Second, the fact that interviews were conducted at the clinic means that other family members, who may have had a role in deciding to donate (or may even disagree with donation), are also excluded. This is potentially significant, given our previous arguments about pets as kin. Given the unexpected themes that emerged around pets as family and proxy donors (see next section), it is also interesting to consider how much further these themes might be developed if longer interviews were to take place in a more familial setting such as the pets’ homes. And third, the lead author stressed that the project was independent academic research but was also open about their own background as a qualified veterinary surgeon, with previous experience of clinical work in a UK pet blood bank. This, combined with the location of the interviews, may have encouraged interviewees to closely associate the project with the veterinary community. This is not necessarily a problem for this particular study, but it would be worth reflecting on whether this could potentially influence research with non‐donors, especially if they felt less able to be critical of blood donation.

## Motivations of animal owners: why do they donate their animals’ blood?

In the following sections we will explore three themes: first, how owners explain their decision as being about saving animal lives, such that the donation is imagined as a ‘gift’ for use by other animals, but also as a contribution to a shared resource which might return a benefit to the donor animal one day. Next we show how the donation experience strengthens and ‘displays’ the kinship between an animal and its owner, and how aiming to protect this bond for other individuals elevates canine blood donations to a virtual transaction between two humans. Lastly, we extend the notion of humans benefiting from canine donation even further by highlighting how some owners’ feelings of guilt or obligation around human blood donation led to them presenting their dogs as ‘proxy donors’.

## Animal benefits in canine blood donation: altruism and mutuality

The primary stated aim in canine blood donation was to save canine lives. The importance of this was heavily emphasised, with some owners being explicit about the lengths they went to in order achieve their aim, such as travelling long distances or taking time off work:

If she can help save one other dog then I think she's doing a good job. It's just cost us a couple of hours but if it, you know, helps save someone else's dog then, you know we're happy. I mean because for us to come here I've taken half a day's holiday from work. That would be my ideal outcome; that she's helped save another dog. (Owner 9)

The opportunity to receive a blood transfusion was constructed as a potential benefit for all dogs including, quite visibly, the donor. Our analysis showed that dog owners, like human blood donors, were strongly motivated by the possibility that their own animal may need a blood transfusion one day:

It's going to benefit other dogs and maybe someday it will benefit him as well … in the future [if] he needs a blood transfusion he might, you know he might get his own back, you never know. If anything happened to him I would be absolutely devastated, you know if a blood transfusion could have saved his life and there wasn't any available. (Owner 3)

The significance of saving a canine life was commonly expressed in terms of the owner's fear of losing their own pet, particularly if a blood transfusion could have saved them. While in the above quote the owner made the unlikely suggestion that the dog might receive his own blood back, most owners clearly recognised that blood from other dogs was likely to be required if their pet were to need a transfusion. This led to a recurring awareness of the need for other pet owners to also be involved, in order to ensure mutual benefits. In this way the pet owners described contributing to a resource which they imagine sharing with other pet owners:

I always think if he had an accident or something and he needed it [blood] and it wasn't there how devastating it would be. I keep thinking what would happen to him if he had a serious sort of ‘dog versus car’, and I would hope that somebody else would have given blood that he could use. (Owner 11).

In these owner extracts, explanations for donation show a close intertwining of benefits for other animals, with the anticipation of a potential for personal return. Returning to the literature on human blood donations, we can see some striking parallels, in that altruism or the giving of a ‘gift’ (Titmuss 1970) appears to feature alongside self‐interested motivations (Bednall *et al*. 2013; Farrugia *et al*. 2010). Furthermore, the overall impression of pet owners wanting to help each other fits closely with the concept of ‘mutuality’ as proposed by Busby (2004). In short, our analysis so far confirms previous sociological findings on human blood donation in terms of what motivates the individual making the donation decision. However, our next theme will deepen and extend this analysis by exploring more indirect aspects of donation, and what this means for our understanding of human‐animal relations and kinship.

## Shared benefits in canine blood donation: human‐animal ‘kinship’

In addition to helping other dogs directly, owners we interviewed also constructed donation as indirectly helping other animal owners. Interviewees were keen to describe the bond they enjoy with their own dog, and were cognisant of the bond between other dog owners and their pets. Canine blood donations were therefore presented as important, not just because they might save a canine life, but because this positive outcome would also have the effect of protecting a human – animal bond:

I feel as though I am doing something. Like I say it might be a child's dog or an old person's companion we might have saved. (Owner 4)

In this example, the owner was even able to identify the particular type of person (child or elder) who might benefit the most because of their strong bond with their pet. In this way we can start to see the emergence of canine blood donation as conceived as a virtual transaction or exchange between two people, where the canine parties involved were not always the central characters in the narrative.

However, while the donated blood was viewed as valuable in protecting human‐animal bonds, the *act* of donation itself was also significant in strengthening or reinforcing the bond between the owner and ‘donor’ animal. For example, some owners reported a feeling of reward gained when the veterinary staff or their friends praised the dog for donating blood. This praise served to strengthen and confirm their own feelings about their pet:

It's nice to have other people appreciate that your dog is, your dog is fabulous, as great as you think he is. (Owner 3)

In other cases, where an owner admitted that their dog was not always well behaved, donating blood appeared to create an opportunity for their bond to be reinforced or protected through a positive experience:

It kind of proves to me that he's as good as I think he is no matter what he does. (Owner 17)

This construction of the donation experience as an event which strengthened the relationship between an owner and their pet was also presented in other ways, such as an opportunity to focus on one dog where the owner had several pets:

It gives me time with him because we have other dogs so we come out together and it's something we do together. (Owner 13)

The owner's accounts often encompassed literal anthropomorphic statements where the owner suggested that their dogs were like humans. For example, one owner described how she treated her dog after he had donated blood:

I do treat him afterwards (…) he now does go to McDonalds, we've been discussing that. (..) Yes it's the only time he has ever been but yeah he goes to Mc Donald's afterwards. And I do treat him like a little baby don't I? (Owner 17)

Some dog owners recognised that this tendency to ‘humanise’ the dogs was likely to have influenced their decision‐making when it came to offering their dogs as donors:

I'm sure we think our animals are humans and you know we kind of put [on] our own perspective, and think that they would think that they wouldn't mind. (Owner 3)

Others used anthropomorphic reasoning to describe and justify the whole concept of canine blood banking, drawing direct parallels between companion animal and human blood donation:

You know how important it is for humans to give blood, so it's got to be the same for dogs as well. (Owner 5)

They need blood as much as we do don't they? (Owner 16)

However, the owners in our study were also very candid about their lack of knowledge about how, when and where the canine blood was actually used:

I have no idea what happens to it [the blood] afterwards, no none at all. (Owner 7)

When pressed, the owners tended to rely upon extrapolations of what they knew about human blood banking systems, making general assumptions about how the blood might be allocated and accessed:

Well I imagine it's like the hospital, it just goes wherever it's needed. (Owner 8)

I assume it's similar to human blood, and it just goes off into some bank on some database. And then if a vet needs it they just sort of take it out and deliver to the vets. (Owner 9)

The ‘kinship’ relationship appeared to mean that even canine members of ‘families’ who donate blood were included by default in such decisions:

Both my husband and myself give blood so why not [our] dogs? (Owner 9)

It's the same as me doing it and it's the same as my husband doing it. Somebody needs it so it's no different for the dog. (Owner 17)

These owners seemed to take pride in their own donation status and saw their pet's ‘donation’ as a natural consequence of their family membership. Indeed, the reverse idea of blood donor pets inspiring their owners to donate blood was recently used by the National Blood Service in an advertising campaign involving UK pop group McFly ‘If my cat can do it then I can’ (NBS 2013). This close association between human and animal donation is interesting, and the interview data also included wider linkages between human and animal donation in the context of the family. For example, one interviewee (Owner 2) twice volunteered detailed information about the tragic death of her son, and their decision to donate his organs. She then linked this to the idea that, just as human organ donation had ‘become natural’ it was likely the same would happen in animal medicine.

This tendency for animal owners to interweave human and animal health and even to translate their animals’ presumed thoughts was also discussed by Ryan and Ziebland (2015) who suggest that anthropomorphic interpretations fail to capture the complex relationship between animal and owner. While we do identify some anthropomorphic accounts, our next section illustrates the simultaneous commodification of donor animals (Fox 2006), which we argue fits closely with the ‘human’ and ‘non‐human’ elements of interspecies ‘kinship’ (Charles 2015).

Our analysis indicates that blood donation is specifically constructed as a family value, or, taking Charles's (2015) view as something that is done as part of kinship. However, what we did not anticipate was the way in which the act of donation is an opportunity to publicly present or ‘display’ this kinship. Finch (2007: 71–3) asserts that ‘the diversity and fluidity of contemporary family relationships are core reasons why displaying families is necessary’ and that ‘relationships need to be displayed in order to have social reality’. Finch (2007: 78) also argues that empirical work is needed to investigate precisely how people convey family. We can now suggest that canine blood donation is one tool through which multispecies kinship is displayed in the health context.

## Human benefits in canine blood donation: doing good by proxy

The owners of dogs donating blood frequently volunteered their own blood donor status; we have already seen how owners who are blood donors used this fact to explain and justify their dog's involvement. What happened with equal frequency was that owners who are not blood donors themselves also used this to justify their attendance at the canine blood donation session:

I haven't actually done it which is why I wanted her to do it because I think it's important that you do something in this world. (Owner 2)

In this case, the dog's donation allowed the owner to fulfil her own need to ‘do something’ positive. We have already suggested that the practice of donating a pet's blood allows for a virtual ‘connection’ to be made between the owners of dogs donating and receiving blood. In some accounts this was made even clearer with the suggestion that even when an owner could not donate themselves, they were helping other humans through donating their pet's blood:

I just never got round to it really … So I feel a bit guilty about that. Yes I do … so perhaps I'm using these as a substitute, as at least they are helping some dogs if not people. Well in the long run it helps people doesn't it because people are so fond of their animals aren't they? (Owner 4)

In this account, the canine blood donation is clearly constructed as a transaction between two humans because it aims to protect the mutual bond they enjoy with their dogs. This account also very clearly expressed the guilt that people claim to feel because they do not donate blood themselves. This guilt appears to be at least partially appeased by the process of donating a pet's blood, and some owners were even more candid about their pet's role in making up for their own deficiencies as failed donors:

I can't give blood because I'm on blood pressure tablets … so I suppose it's a bit like doing good by proxy isn't it? You make the dogs do it instead. (Owner 14)

Furthermore, when a dog did not successfully donate blood because it was unsuitable for medical or behavioural reasons, the owners appeared to find it difficult to accept the loss of this opportunity:

I think it is disappointing because I've, because of Tess and because of losing Fred as well, because I've had some you know tragic circumstances in my life I would have liked to have had one [animal] that was able to help others. But maybe the next one. (Owner 21)

In this case the owner had built up some considerable pressure upon herself and her dog because she felt she needed to ‘repay’ the pet owning community for help she had been given when her previous pets were ill. These narratives were commonly expressed by participants, where canine blood donation functioned as an opportunity for the owner to ‘repay’ acts of kindness, either within the context of pet ownership or within human society and medicine at large. What this means for wider sociological understanding of health will now be discussed.

## Discussion and conclusions

This study is empirically novel in focusing on companion animal blood donation. Our overall aim was to use interviews with pet owners who donate their animal's blood to better understand what constitutes motivation in this emerging medical field. The article used existing literature on human donation and human‐animal relations to relate a framework of human‐animal ‘kinship’ (Charles 2015) to sociological ideas of altruism/mutuality, kinship/display, and medical ethical concepts of consent/proxy decision‐making.

The first theme confirmed the robustness of previous work on human blood donation: beyond altruistic motivations of helping others by providing a gift, the data analysis revealed the role of self‐interest and mutuality (Busby 2004) in explaining why owners donate their animals’ blood. However, our second theme went further to show how donation was also claimed to benefit human owners through protecting the human‐animal bond which can be understood if pets are recognised as kin (Charles 2015). We then extended Charles’ argument to show how owners are in fact using donation as an opportunity to display (Finch 2007) and reinforce this multi‐species kinship. This argument contributes significantly to sociological work on human‐animal relationships, more on which below. Finally, our third analytical theme explored how owners use canine blood donation to relieve their own feelings of guilt or obligation through ‘doing good by proxy’. We now reflect on the wider implication of these findings for the sociology of (human) donation, human‐animal relations, professions involved in canine blood donation, and finally for wider sociological understandings of healthcare.

This article has several implications for the sociology of donation. A crucial finding is that some of the ‘psychological’ benefits which have been identified for human blood and tissue donors, such as increased self‐worth or alleviation of obligation, appear to be experienced by those consenting to a donation from their pets. This indicates that the positive feelings traditionally assumed to be associated with ‘donation’ can be separated from the physical act, and are ‘transferred’ to a third party who is providing consent. This raises considerable ethical questions and challenges sociologists of donation to ensure that their research is sufficiently open to capture these complexities. We also hope that our claim that donation is used as an opportunity for family (kinship) ‘display’ (Finch 2007) and for doing good by proxy will be critically evaluated by those working in the sociology of (human) donation. At a minimum, this study adds weight to the move (Taylor‐Sands 2015) towards considering the family rather than the individual as the donating unit. More radically, our claims raise difficult questions concerning how guilt is experienced and managed, and, how the ideal of family is socially enacted in the medical setting.

For colleagues interested in human‐animal relations, this study is useful in supporting those models which stress the simultaneous role of pets as both companions and possessions (Charles 2015, Fox 2006). In our case, the animal is even more complex – at once a potential patient, a loved family member which whom to bond, and a route through which a kind of health citizenship (by doing good) is acheived. This latter argument necessitates futher conceptual work and should encourage much more attention to animal medicine as an important site where interspecies relationships are made and displayed, and where nature/culture, animal/human boundaries (Fox 2006) are potentially transgressed. Further work could re‐examine canine blood donation from the point of view of animal subjectivity, or the human‐animal hybrid (Haraway 2007). However, we argue that retaining division and difference (Latimer 2013) is important, and potentially allows more space for a normative critique of the benefits (or otherwise) for animals of such technological innovations.

Indeed, for canine blood donation clinics, our analysis represents a potential challenge to their current practices. For example, it may be that owners need more information about the realities of accessing blood for their own animals, and thus whether their assumptions about mutuality are practically possible. Clinics therefore need to reflect on the way owners extrapolate from their knowledge of the human National Blood Service, and whether this is ethically appropriate. The article also serves as a particularly dramatic example of the wider responsibility challenges facing the veterinary professional (see Hobson‐West and Timmons 2016) whose primary task, according to the declaration all graduates make (RCVS 2014) is to prioritise animal welfare, yet who may be called on to facilitate practices such as blood donation which are not directly beneficial for the individual animal but which appear to create significant human benefit. In short, might such practices create the risk of conflict between the interests of the ‘donor’ animal and its owner? If so, this raises critical ethical questions concerning power and authority in the veterinary setting.

And finally, this article has important implications for the sociology of health and illness more widely by graphically demonstrating the close interrelationship between human and animal health care. Just as pets are important actors in patients’ individual illness narratives (Ryan and Ziebland 2015) so we have also found that donation narratives tend to entangle human and non‐human animals. So what if this is the case for other (more mundane) domains of contemporary medical practice, thus far overlooked due to the power of the ‘species barrier’? (Charles 2015). Put simply, what if individuals’ everyday healthcare decisions or rationales are influenced by experiences in the animal clinic, and vice versa? This opens up an entire new vista of research for sociologists, where species becomes one of the elements we critically study, rather than a fixed barrier at which our analysis of health and medicine stops.
